# Tuning the physicochemical features of titanium oxide nanomaterials by ultrasound: Elevating photocatalytic selective partial oxidation of lignin-inspired aromatic alcohols

**DOI:** 10.1016/j.ultsonch.2023.106306

**Published:** 2023-01-21

**Authors:** Abdul Qayyum, Dimitrios A. Giannakoudakis, Dariusz Łomot, Ramón Fernando Colmenares-Quintero, Alec P. LaGrow, Kostiantyn Nikiforow, Dmytro Lisovytskiy, Juan Carlos Colmenares

**Affiliations:** a Institute of Physical Chemistry, Polish Academy of Sciences, Kasprzaka 44/52, 01-224 Warsaw, Poland; b Faculty of Engineering, Universidad Cooperativa de Colombia, Medellín 50031, Colombia; c Scientific Imaging Section, Okinawa Institute of Science and Technology Graduate University, Kunigami-gun, Okinawa 904-0412, Japan

**Keywords:** Ultrasound assisted precipitation synthesis, Titanium oxide nanostructure, Photocatalysis, Benzyl alcohol selective oxidation, Biomass valorization

## Abstract

The research for “green” and economically feasible approaches such as (photo)catalysis especially for biomass valorization such as selective oxidation of biomass derived compounds like aromatic alcohols to corresponding aldehyde by avoiding the harsh reaction conditions and the addition of reagents concentrate the focus of attention the last years. Hence, design and development of novel photocatalyst for the partial selective oxidation is highly desirable. In this research work, ultrasonication of different frequencies (22, 40, 80 kHz) and different amplitudes was utilized as synthesis tool in order to obtain novel materials by precipitation method. The synthesized samples were characterized by using different techniques such as N_2_ sorption, TEM, XPS, XRD, thermal analysis, and diffuse reflectance spectroscopy. The synthesized sample by using low ultrasound frequency (22 kHz) and amplitude showed a mixed morphological and structural nature consisting of asymmetric 1-dimensional (nanorods-like), layered nano-structures and not well-defined areas, leading to elevate for metal oxide specific surface areas up to 155 m^2^/g. The observed 1-D nanostructures have diamentions in the range of 20–60 nm. This sample revealed the highest photo-oxidation efficiency for the selective conversion of two biomass-derived, and more specifically lignin-inspired model compounds, benzyl alcohol and cinnamyl alcohol to benzaldehyde and cinnamyl aldehyde, respectively, and hence the highest yield towards the desired aldehydes. The selective photo-oxidation activity was retained even after 5 photocatalytic cycles, while no leaching of Ti was recorded.

## Highlights

• The ultrasound was successfully utilized as a mean of precipitated synthesis of TiO_2_.
• The ultrasound assisted synthesized TiO_2_ samples showed the formation of nanostructures.
• The optimization of utilized frequencies and different amplitudes is necessary to alter/tuned the photoactivity.
• The additive free photocatalytic selective oxidation of aromatic alcohols to corresponding products was studied.
• Our novel ultrasound assisted synthesized TiO_2_ showed higher selectivity of products.

## Graphical abstract

**Figure:**
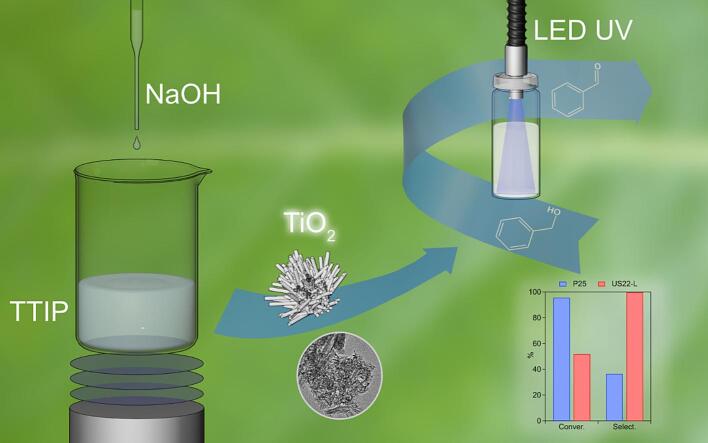

## 1 Introduction

**Scheme 1:**
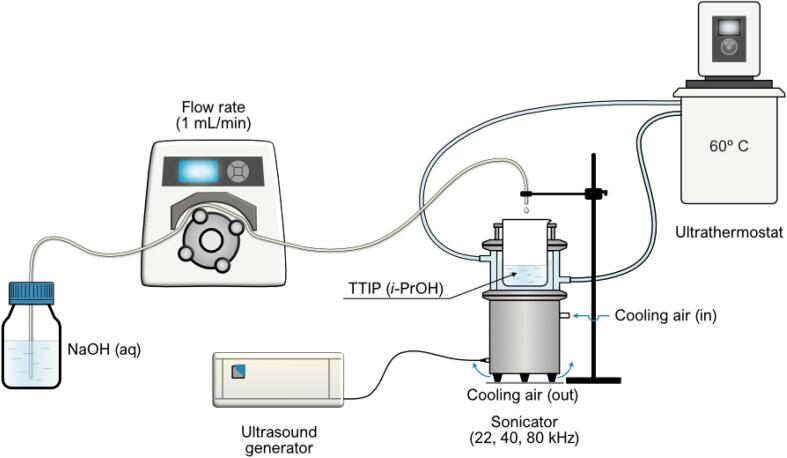
Schematic illustration utilized for the entire synthesis setup.

The selective transformation of aromatic alcohols is a fundamental and very important reaction both for basic lab research and practical mass-production processes. Aromatic alcohols can be selectively converted to high-value compounds such as corresponding aldehyde, ketone and carboxylic acids [1], with many of them to be important for industrial processes. For instance, the synthesis of benzaldehyde (PhCHO) and cinnamyl aldehyde (CinAld) by selective oxidation of benzyl alcohol (BnOH) and cinnamyl alcohol (CinOH) is of great importance since benzaldehyde is used as intermediate for the synthesis of various fine chemicals used in various industries such as medicine, perfumes, cosmetics, food and plastic additives etc. [2], [3]. In general, a plethora of approaches have been adopted for selective oxidation reactions. In the past few decades, various traditional approaches, such as the chemical oxidation methods, have been adopted for the partial selective transformation of BnOH to PhCHO. But, these methods demand oxidizing agent such as permanganate, dichromate, chromic acid, chromium trioxides in a stoichiometric amount leading to expensive and toxic in nature processes [1], [4], [5]. Additionally, these traditional approaches suffer from harsh reaction conditions, low controllability, and environmental harm, leading to remarkable serious environmental footprint and energy waste [6], [7]. To overcome these drawbacks, it is highly desirable to develop a green, sustainable and more efficient method for the selective transformation of BnOH to PhCHO (see Scheme 1 and Table 1).

**Table 1:** The followed ultrasound parameters and the abbreviations of the synthesized materials.

Serial No.	Used ultrasonic frequency (kHz)	US Amplitude (µm)	US Power (W)	Samples abbreviation
1	22	10	8	US22-L
2	22	50	40	US22-H
3	40	30	15	US40-L
4	40	70	35	US40-H
5	80	30	15	US80-L
6	80	70	35	US80-H
7	–	–	–	MagS*

*Magnetic stirring (200 rpm).

Heterogeneous photocatalysis has attracted increasing interest as an alternative to the chemical oxidation approach, since it can have a more environmental friendly character [8], [9], [10]. Semiconductors based photocatalysts have been proven potential applications in various fields such as environmental remediation, hydrogen production, CO_2_ reduction etc. [11], [12], [13], [14]. Nanostructured TiO_2_ has been extensively explored for a range of photocatalytic applications among since Fujishima and Honda’s pioneering work on photoelectrochemical water splitting using TiO_2_ in 1972 [15]. TiO_2_ based materials are considered as benchmark materials due to possessing unique properties, such as low toxicity, high chemical and thermal stability [16]. TiO_2_ based nanomaterials are widely used for environmental remediation applications to decompose organic pollutants, but their use in the selective transformation of organic compounds is limited due to their uncontrollable/unselective high photoreactivity. Tuning the physicochemical properties such as the textural, morphological and surface chemistry heterogeneity has a unique role on manipulate the reactivity. The method of synthesis can determine the physicochemical properties and hence novel TiO_2_-based materials and synthetic protocols are still of a high interest [17].

Various synthesis methods such as solvothermal, sol–gel, hydrothermal etc. have been adopted to synthesize TiO_2_-based nanomaterials [18]. However, some limitations exist, such as low specific surface area, low porosity, and low selectivity for the targeted compounds, which led to limiting the use of TiO_2_ as photocatalysts for organic synthesis. So, the design of novel TiO_2_ that can overcome these limitations and targeting on specific materials for specific photocatalytic reaction is still an ongoing and challenging research task. Sonochemistry is a rapidly growing field that takes advantage of ultrasound power and cavitation phenomenon resulting in altered physical and chemical effects [19]. The use of sonochemistry in material synthesis has gained great attention due to various advantages such as of being greener, environment-friendly, energy-efficient, and with short duration of ultrasound (US) exposure novel and unique nanomaterials can be derived [20]. The major role of ultrasound in the synthesis step is attributed to many phenomena derived from the adiabatic generation and growth of acoustic cavitation leading to enhanced mass transformation effect, de-aggregations effects, the formation of localized hot spots (up to 4726 °C and 500 atm), and the formation of free radicals [21]. By controlling the frequency and power of US, it is feasible to manipulate on-demand the physicochemical features of synthesized materials [22], [23], [24], [25].

The key goal of this research work was to study the effect of different frequencies of US irradiation with varying also the US power during the synthesis of novel TiO_2_ nanostructures by precipitation and to determine how the use of US of different frequencies and amplitudes/powers affected the physicochemical features of the final synthesized photocatalysts. The strategy was established considering to be green, sustainable and of a low cost since the final yields were expected high while the volume of wastes is minimal. For this research work, three different US frequencies were applied, specifically 22, 40 and 80 kHz, at two different powers.

A sample of TiO_2_ was also synthesized by using magnetic stirring instead of US for the study of overall effect of US irradiation on the final physicochemical properties as well as the photoactivity of synthesized TiO_2_ materials. A commercially available benchmark sample, namely TiO_2_ P25 was also studied for the sake of comparison. The ultimate goal was to obtain a photocatalyst of high selectivity and yield towards additives/reagents-free partial selective oxidation of BnOH to PhCHO under low-power light irradiation at ambient conditions and to explore how the altered physical and chemical parameters play a key role.

## 2 Experimental part

### 2.1 Materials

The chemicals and materials used were titanium isopropoxide (TTIP, 98 %, Acros Organics), isopropanol (99.7 %, POCH), sodium hydroxide (NaOH, ChemPure), benzyl alcohol (BnOH, 99.5 %, ChemPure), benzyl aldehyde (PhCHO, 99 %, ChemPure), cinnamyl alcohol (98 %, Acros Organics), cinnamyl aldehyde (≥98 %, Roth), acetonitrile (AcN, HPLC grade, POCH) and TiO_2_ (P25, Evonik Degussa). All the chemicals were used as received.

### 2.2 Synthesis of the catalysts

The synthesis of novel TiO_2_ was performed by the assistance of ultrasonication using precipitation method [25]. For the synthesis, a solution of titanium isopropoxide and isopropanol with the volumetric ratio of 1:3 was prepared and 20 mL of this solution was taken in a beaker (250 mL volume). This beaker was kept inside the Cuphorn sonicator (Sinaptec, NexTgen Lab500 generator) .100 mL of the an aqueous solution of sodium hydroxide (NaOH, 2 M) was dropwise added with a control flow rate (1 mL/min) by using peristaltic pump (Master Flex L/S, Cole-Parmer). The temperature of water bath of sonicator was stabilized at 60 °C during the sonication by using the thermostat (ultrabath Julabo DB-5 (Corio CD-B5)). After the addition of 100 mL of NaOH solution, the white (colloidal) suspension was obtained which was filtered by using Whatman paper (Rotilabo®-Round Filter) followed by washing with dilute hydrochloric acid (0.1 N) and water to get the neutral pH of obtained filtrate.

The powder TiO_2_ sample was obtained after drying this filtrate at 90 °C for 16 h in an oven under static air. The above synthetic protocol was followed for the synthesis of a series of materials by altering the frequency and amplitude or power of the irradiated ultrasound. Three different frequencies with different amplitudes {22 kHz (10 and 50 µm), 40 kHz (30 and 70 µm) and 80 kHz (30 and 70 µm)} were used during the synthesis of TiO_2_ samples. The total power of 22 kHz sonicator was different than 40 and 80 kHz sonicators, so the nearest possible amplitudes of 22 kHz comparing to 40 and 80 kHz sonicators was used for the synthesis.

In order to study the effect of ultrasonication on the material synthesis, a sample of TiO_2_ was also synthesized with the help of magnetic stirring (200 rpm) without utilization of ultrasound while keeping same all above precursors and protocol. This material is referred to as MagS.

### 2.3 Physicochemical characterizations

Nitrogen physisorption measurements were performed by a Micromeritics instrument (model ASAP 2020) at −196 °C. Before the measurements, the samples were degassed under vacuum at 90 °C for 6 h. Brunauer Emmett–Teller (BET) method was used for the calculation of specific surface area (S_BET_) from the obtained isotherms, while the pore volume and size were estimated by the Barret-Joyner-Halenda (BJH) approach [26], [27].

Diffuse reflectance (DR) spectra were collected from UV-B (250 nm) to infrared (900 nm) range by the instrument Jasco V-570 spectrophotometer equipped with the integrating sphere (Spectralon). The baseline was obtained with poly(tetra- fluoroethylene).

Transmission electron microscopic (TEM) images was obtained on a JEOL JEM 2100 operating at 200 kV with a LaB_6_ filament. TEM samples were prepared by sonicating the nanoparticles into ethanol and then depositing them onto 200 mesh carbon coated copper grids (Ted Pella, Inc). The powder X-ray diffraction (XRD) patterns were obtained using a Siemens D5000 diffractometer (40 kV and 40 mA) equipped with a horizontal goniometer. The weight loss was measured by the thermal gravimetric analysis by using thermos-balance (Mettler-ToledoTGA/DSC3 +).

The surface chemistry heterogeneity was studied by using X-ray photoelectron spectroscopic (XPS) method which were performed by using PHl 5000 VersaProbe™ Scanning ESCA Microprobe (ULVAC-PHI; Chigasaki Japan) instrument. XPS spectra were obtained by using monochromatic Al-Kα radiation (hν = 1486.6 eV), while the X-ray source was operated at 15 kV, 25 W and 100 µm spot size. Whereas the high-resolution (HR) XPS spectra were obtained by an analyser pass energy of 23.5 eV and an energy step size of 0.1 eV. The analysis of survey spectra was performed with the PHI Multipak software. The deconvolution and quantification of obtained spectra was performed by Casa XPS software (v.2.3, Casa Software ltd, Wilmslow, United Kingdom).

### 2.4 Photocatalytic activity evaluation tests

The additive free photocatalytic performance of TiO_2_ catalysts was evaluated following the photocatalytic partial selective oxidation of benzyl alcohol (BnOH) to benzyl aldehyde (PhCHO) and cinnamyl alcohol (CinOH) to cinnamyl aldehyde (CinAld) and PhCHO. A commercially available TiO_2_, P25 Evonik, was used as a benchmark for the sake of comparison. For each photocatalytic experiment, 15 mg of each sample were added in a reactor (25 mL) which contained 15 mL of substrate solution (1 mM in acetonitrile) under magnetic stirring (600 rpm). The reactor was kept inside a water bath at the stabilized temperature of 30 °C using thermostat (ultrabath Julabo DB-5 (Corio CD-B5)). The reactor was cover with the aluminium foil to avoid any exposure of external light.

Photocatalysis performance was studied under ultraviolet light irradiation (UV, 365 nm) produced by light-emitting diodes (LEDs) (LEDMOD.V2, Omicron, Germany). Prior to light irradiation, the mixture was stirred in the dark for 1 h for equilibration/stabilization. After that, the lamp of the specific irradiance of 100 W/m^2^ (measured by a radiometer Delta OHM, HP2302.0) was switched on. Samples (0.2 mL) for analysis were collected by a Teflon tube (diameter 0.7 mm) and a syringe (2 mL) after the equilibration in the dark (0 point) and after specific time intervals of light exposure and then immediately filtered by filter (0.20 µm pore size).

The analysis of collected samples during the photocatalysis tests were performed by Gas Chromatograph (Shimadzu GC-2010) which was equipped with a flame ionization detector. A capillary column (Zebron ZB-5MS, Phenomenex USA) of 30 m length, 0.25 mm inner diameter and 0.5 μm film thickness was used and He was chosen as the carrier gas. This analysis was performed by injecting 1 μL of the sample by using split ratio of 8.

For GC analysis of samples collected from the photocatalytic experiments, the temperature of the column was set at 50 °C for initial 3 min and, then increased up to 300 °C with a ramp of 9 °C/min and the final holding time was established 2 min. The photocatalytic efficiency evaluations were based on BnOH and CinOH conversion, PhCHO and CinAld yields and selectivity, and mono-aromatic balance which were calculated by using the following equations:

(1) $$Conversion\left(\%\right)=\left[\frac{\mathrm{Co}-Cr}{\mathrm{Co}}\right]*100$$

(2) $$Yield\left(\%\right)=\left[\frac{\mathrm{Cp}}{\mathrm{Co}}\right]*100$$

(3) $$Selectivity\left(\%\right)=\left[\frac{\mathrm{Cp}}{\mathrm{Co}-Cr}\right]*100$$

$\mathrm{AromaticBalance}\left(\%\right)=\left[\frac{\mathrm{Cr}+Cp}{\mathrm{Co}}\right]$*100 (4).

Where Co is the initial concentration (mM) of substrate, while Cr and Cp, are the concentrations of the substrate and the targeted product after the specific time interval of the reaction.

## 3 Results and discussion

**Fig. 1:**
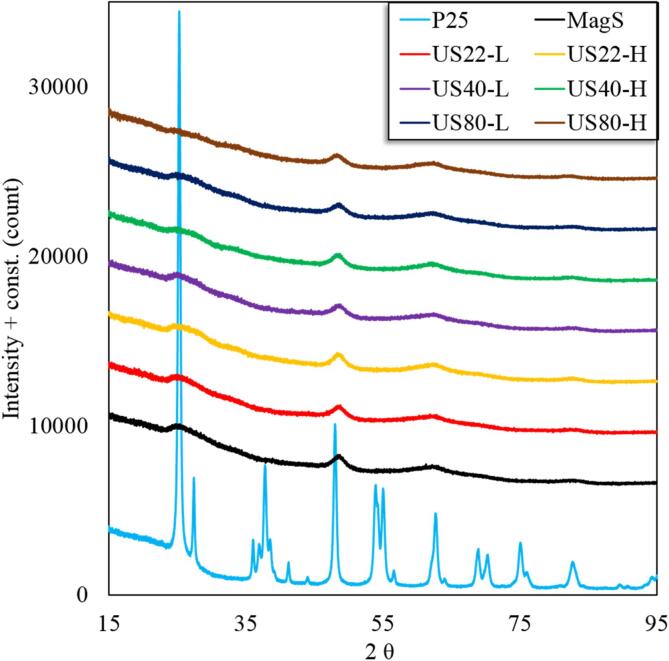
XRD patterns of all the synthesized materials and TiO_2_ P25.

The crystallographic structure of the all the synthesized as well as commercial samples was investigated by powder XRD. The obtained diffraction patterns are presented in Fig. 1. All the synthesized materials showed a predominately an amorphous and/or nanosized nature. The synthesized materials possessed three peaks at 25, 47 and 63° 2θ, which are the characteristic of the anatase phase [28], [29]. Although, the low intensity and the high broadness of these peaks suggested that the anatase crystals are of nano size and/or of low percentage compared to the entire material.

**Fig. 2:**
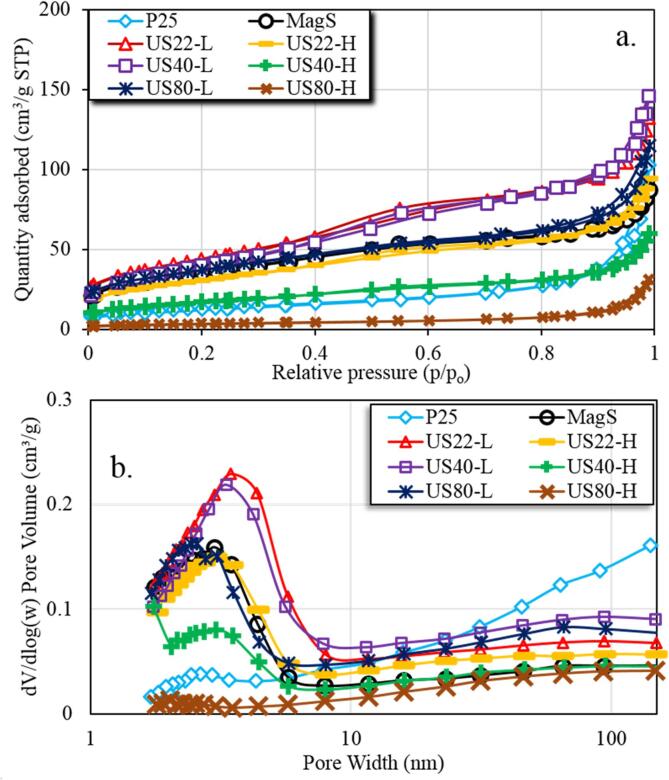
N_2_ sorption measurement for P25, MagS and US assisted synthesized TiO_2_ (a) N_2_ adsorption–desorption isotherms based on BET method (b) pore size distributions based on BJH method.

N_2_ sorption characterization was performed to evaluate the textural properties of all the synthesized, and commercial TiO_2_ and the derived outcomes are presented in Fig. 2 and Table 2. The first derived outcome is that the utilization of low power ultrasonication results in the formation of porosity, with all the materials synthesized at low energy US to have higher values of the textural features compared both to the material MagS, as well as compared to the commercial nanoparticles TiO_2_ P25. More interestingly, the increase of the ultrasound (US) power has an intense negative impact on the textural properties, especially for the 80 kHz. The sample synthesized under the irradiation with the US of the lowest frequency (22 kHz) with low power, referred as US22-L, showed the highest values of textural features. The specific surface area (S_BET_) was 155 m^2^/g while the total pore volume (V_Tot_) 0.192 cm^3^/g, values that are around 25 and 50 % higher compared to MagS. The sample synthesize under irradiation of low-power 40 kHz showed the second better value of specific surface area (S_BET_) = 141 m^2^/g but on the contrary presented the highest total pore volume equal to 0.209 cm^3^/g.

**Table 2:** Textural and optical parameters of all the herein studied materials.

**Index** **numb.**	**Material**	**Specific surface area** **(m^2^/g)**	**Pore volume** **V_p_ (cm^3^/g)**	**Pore size** **d_p_ BJH (nm)**	**Estimated band gap** **(eV)**	**Surface pH**
1	P25 (Evonik)	46	0.138	13.7	3.21	6.1
2	MagS	124	0.127	4.4	3.45	7.9
3	US22-L	155	0.192	4.9	3.45	8.1
4	US22-H	108	0.135	5.1	3.47	8.4
5	US40-L	141	0.209	11.2	3.44	7.9
6	US40-H	60	0.085	5.8	3.57	8.5
7	US80-L	131	0.163	5.9	3.52	8.3
8	US80-H	12	0.041	12.6	3.57	8.9

For the US22-L and US40-L synthesized materials, the isotherms revealed to be a complex shape which suggested the presences of microporous and mesoporous features. Whereas all other synthesized materials, the shape of the isotherms are closed to type I(b) and II based on the IUPAC report [30], which suggested that the synthesizes materials have pores containing the micropores (1.6 to 2 nm) and also mesopores (2.5 to 9.5 nm) which can be seen on pore size distribution (Fig. 2b). Based on these results, it can be concluded on the regards of utilizing US as a synthetic tool that both frequency and power matter and should be optimized during the development of novel nanomaterials by precipitation.

**Fig. 3:**
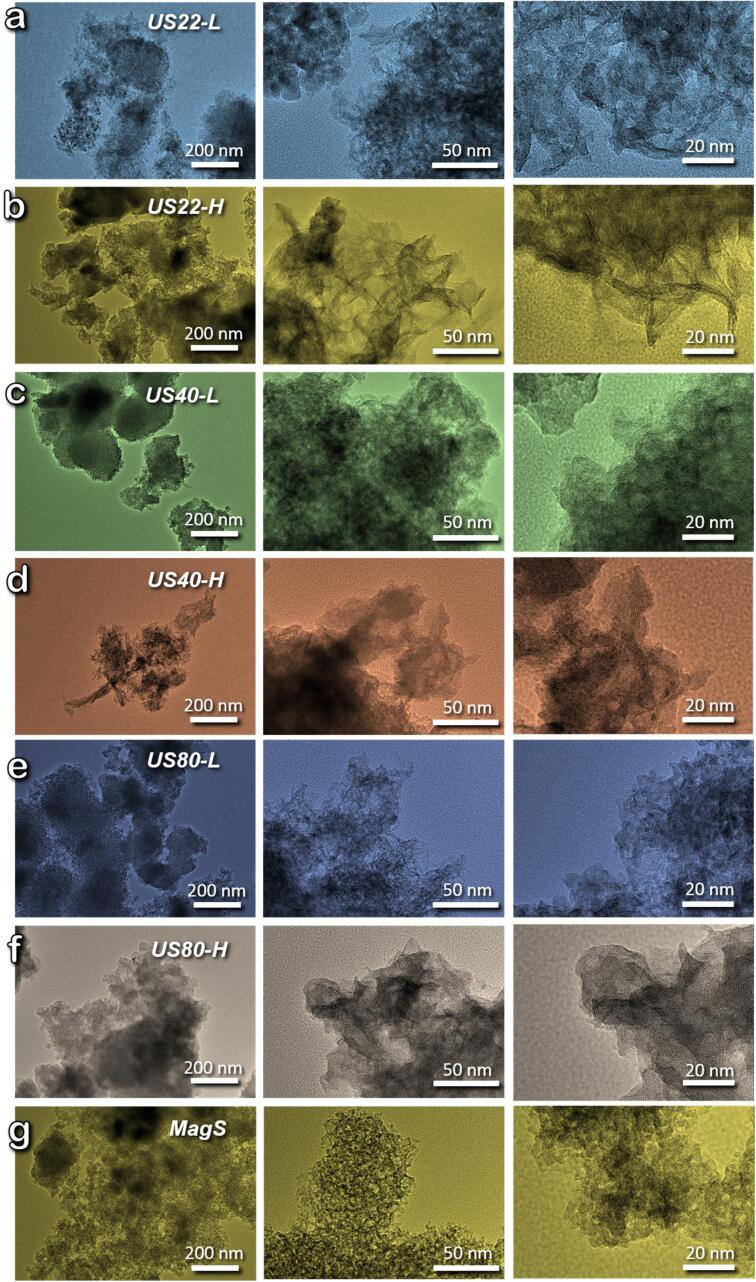
TEM images of synthesized samples.

Since we observed that the use of different US frequencies and powers during the synthesis step alters the textural features of the synthesized titanium oxide, the morphological nature of the samples was also explored. Fig. 3 collects the TEM images of all synthesized materials. For all samples, a nano-scaled morphology can be observed, in accordance with the XRD analysis. In all samples except for the material obtained under the silent conditions (MagS), one dimensional growths were observed with an interlayer spacing of ∼7 Å, which is indicative of the structure of titanium oxide/titanate nanorods_._ The US assisted samples obtained by using 22 kHz showed the formation of the most distinctive nanostructures (Fig. 3a,b), which are better shaped in the case of high power, with length ranging from 20 to 60 nm and distinctive interlayer spacings (Fig. S1a,b). For the samples obtained using either 40 or 80 kHz (with both powers), no specific morphology was observed (Fig. 3 c-f). Although the formation of 3-D aggregations consisting of particles with the ∼7 Å interlayer spacings can be seen. The material obtained under silent conditions (MagS) did not show the formation of nanorods, since spherical shaped nanoparticles in the range of size 2–8 nm were observed (Fig. 3g), and the distinctive interlayer spacings were not observed, instead at least one of the spherical particles observed could be indexed to the crystal structure of anatase (Fig. S1c).

**Fig. 4:**
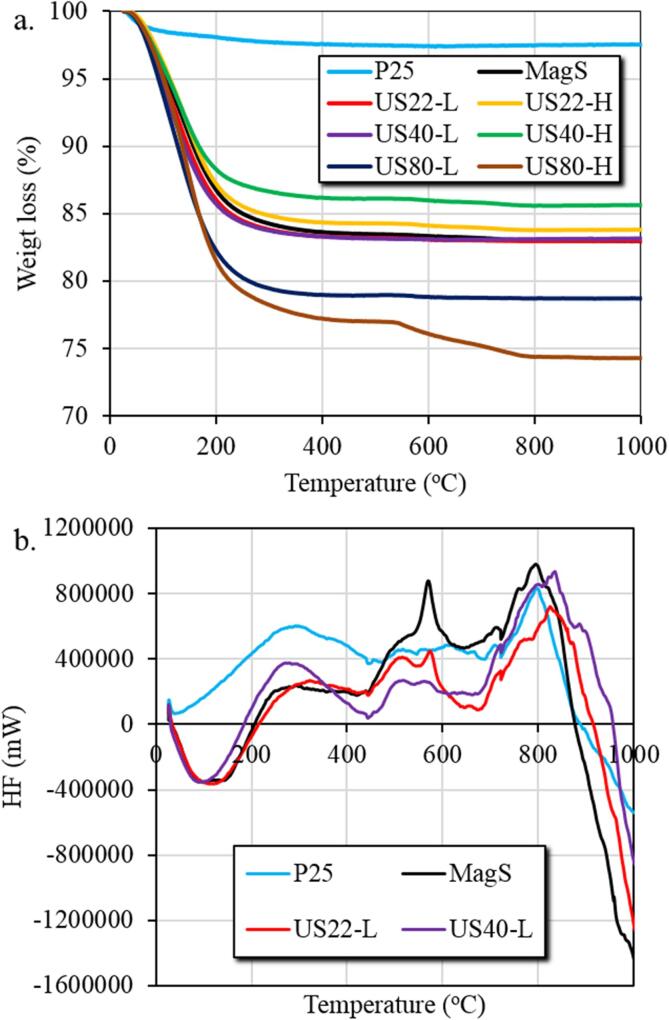
(a) Thermograms (b) differential thermal analysis for P25, MagS and US assisted synthesized TiO_2._

The thermal analysis of all the tested samples was also performed in order to study the weight loss upon increase in the temperature. The commercial TiO_2_ showed a very less weight loss of 2.4 % till the 400 °C. On the contrary, all the synthesized samples showed higher weight loss up to 400 °C (Fig. 4a). US22-H and US40-H showed lowest weight loss 15.5 % and 13.8 %, respectively. The other synthesized samples such as MagS, US22-L, US40-L, US80-L showed weight loss of 16.4 %, 16.7 %, 16.7 % and 21.1 %, respectively, while the highest weight loss of 22.8 % was found for the US80-H sample. The highest weight loss showed by all the synthesized samples suggested the presence of higher amount of hydroxyl groups on the surface.

Differential thermal analysis (DTA) of all the synthesized and commercial TiO_2_ samples was studies in order to evaluate the phase transformation kinetic of the amorphous phase. DTA measurements (Fig. 4b, Figure S2) showed different peaks in different ranges of temperature. The first peak only for the synthesized TiO_2_ samples appeared in the range 0–200 °C that represents the endothermic process which indicate the evaporation of residual water moieties and other organic residues [31], [32]. But this peak did not appear for P25 as it is synthesized with pyrolysis method at high temperature. Another peak appears in the range 200–450 °C for all the synthesized samples except US80-L that represent the exothermic process which indicate the removal of other organic residues from the TiO_2_ samples and the broadness of this peak indicate the crystalline phase [33], [34]. The strong peak in the range 500–600 °C indicated the exothermic process of amorphous to anatase phase, MagS sample showed more intense peak in the range 500–600 °C which is the indicative of crystal structure of anatase and is in good agreement with the HRTEM image. While the peak appear in the range 750–900 °C might be indicated the anatase to rutile phase transformation [35].

The X-ray photoelectron spectroscopy analysis (XPS) was carried out in order to study the differences on the surface of all the herein studied samples. The atomic percentage obtained from XPS core energy level analysis of O 1 s, C 1 s, Ti 2p and Na 2p for all the synthesized samples and commercial P25 samples is presented in Table 3, while the corresponding high resolution deconvoluted spectra are presented in Figure S3-S4. The first outcome from the surface chemistry analysis is that all the synthesized samples contained the sodium on their surface, whereas the commercial P25 sample did not possessed sodium. The presence of Na may be due to the adopted synthesis procedure, since sodium cations play a key role on the formation of 1-D titanate nanostructures [36]. Due to spin–orbit coupling, Ti 2p spectra present as a doublet (Ti 2p 3/2 and Ti 2p 1/2 peaks separated by approximately 5.7 eV. Position of the Ti 2p 3/2 for all the samples is around 458.8 eV BE which is in the agreement with the reported titanium oxide (IV) [37], [38], [39])_._ The high-resolution deconvolutions spectra of O 1 s for all samples showed two peaks, one in the range of 529.9–530.5 eV, which represents the bridging oxygen of the metal oxide matrix (Ti-O-Ti) whereas the second peak appeared in the range of 531.2–532.1 eV, which correspond to the adsorbed oxygen such as hydroxyl radicals on the surface of the TiO_2_ samples [40], [41]. The deconvoluted spectra of C 1 s core energy of all the samples revealed three peaks, the first one in the ranges of 284.6–284.9 eV confirming the presence of C—C groups, the second one at the 286.0–286.8 eV representing the C—O—C groups, whereas the third one appeared in the ranges of 288.8–289.6 eV linked to the presence of C=O groups [37], [42].

**Table 3:** The atomic % obtained from XPS core energy level analysis of O1s, C 1 s, Ti 2p and Na 2p for all the synthesized samples and commercial TiO_2_ P25.

	**O1s**	**O1s**	**Ti 2p 3/2**	**Ti 2p 1/2**	**C 1 s**	**C1s**	**C 1 s**	**Na 2p**
	**(Ti-O-Ti)**	**(O**—**H)**			**(C**—**C)**	**(C**—**O**—**C)**	**(C**=**O)**	
								
	530.72	531.99	459.18	464.88	284.8	286.17	289.58	31.53
P25	58.84	4.38	17.18	8.58	8.76	1.08	1.18	---
MagS	57.75	4.08	16.26	8.12	2.66	0.45	0.85	9.83
US22-L	54.8	7.60	16.72	8.35	2.26	0.72	0.7	8.85
US22-H	55.63	4.15	16.24	8.11	3.34	0.45	0.67	11.41
US40-L	53.7	5.16	16.56	8.27	2.49	0.47	0.46	8.96
Us40-H	54.97	3.72	15.57	7.78	3.87	0.64	0.8	12.65
US80-L	56.39	4.57	16.26	8.13	2.91	0.48	0.65	10.61
US80-H	52.86	4.43	15.17	7.58	3.97	0.54	1.29	14.16

**Fig. 5:**
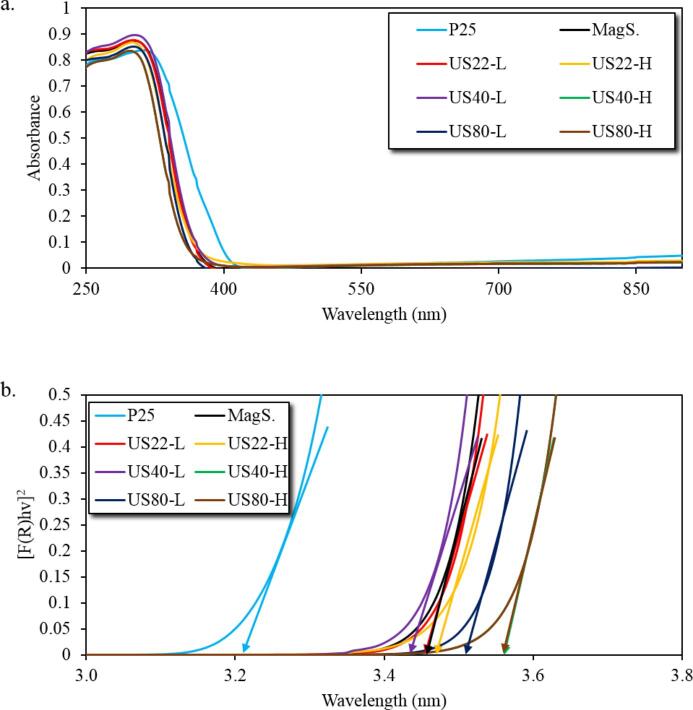
(a) Diffuse reflectance absorption spectra (b) Tauc plots for P25, MagS and US assisted synthesized TiO_2._

The optical features were also evaluated based on defuse reflectance method in the range from 250 to 900 nm. The absorption spectra (Fig. 5) revealed that all the synthesized samples did not reveal light absorption in the boundaries between UV-A, and visible light (380–410 nm) as P25 did. The optical band gaps (E_g_) were estimated by using the Tauc plots method which derived based on the Kubelka–Munk function [43], [44], which were found in the range from 3.44 to 3.57 eV for the synthesized materials, which are slightly higher although close as compared to P25 (3.21 eV) and within the ultraviolet range of light (Table 2). It is worthy to point out that the low power US led to slightly lower E_g_, independently the frequency.

### 3.1 Photocatalytic experiments

The performance of the synthesized TiO_2_ was studied by the photocatalytic partial selective oxidation of benzyl alcohol (BnOH) to benzaldehyde (PhCHO) and cinnamyl alcohol (CinOH) to cinnamyl aldehyde (CinAld) and PhCHO without the addition of any oxidative agent. The potential reactions was presented in Fig. S5a,b. The commercially available TiO_2_ (P25) is well known photocatalyst used for the degradation of organic pollutants but this degradation is unselective transformation. For the photocatalytic conversion of organic compounds to the targeted products, high selectivity and hence yield towards the desired compound are uppermost desirable.

We must point out that the ultimate goal upon the photocatalyst design and development is to be able photocatalytically to oxidize BnOH and to form PhCHO. Off course, and to have an elevated ability to convert BnOH is important, but this aspect can be improved by altering and optimizing the catalytic setup and tuning specific parameters, as for instance increasing the temperature of reaction, adding additionally (redox) agents and even utilize the material in continuous flow systems.

**Fig. 6:**
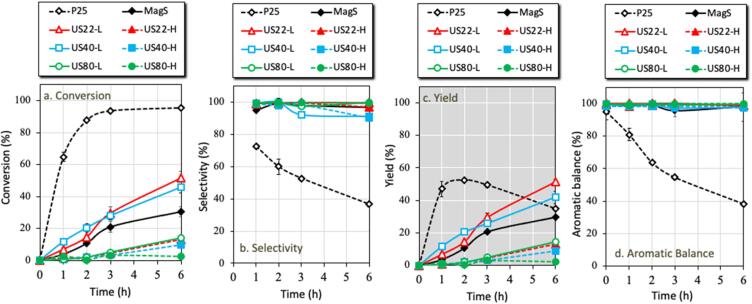
Photocatalytic activity of P25, MagS and US assisted synthesized TiO_2_, (a) conversion of BnOH, (b) selectivity of PhCHO, (c) Yield of PhCHO and (d) aromatic balance of the reaction.

Even though P25 showed a very elevated BnOH conversion, its capability to unselectively convert also the desired aldehyde is undesirable, since it has a negative effect of the process yield. With numbers, P25 reached the maximum BnOH conversion of 93 % within 3 h of UV irradiation. The selectivity started from the beginning at 73 % after the first hour but showed a continuous decrement trend, reaching after 3 h at 53 % and so the yield was 49 %. Since no other aromatic compounds were detected, the conversions of both BnOH and PhCHO are assumed as highly unselective (Fig. 6). And this is an important drawback, since the formation of small organic compounds or/and dimers will negatively affect the last stage of synthesis, the purification.

**Fig. 7:**
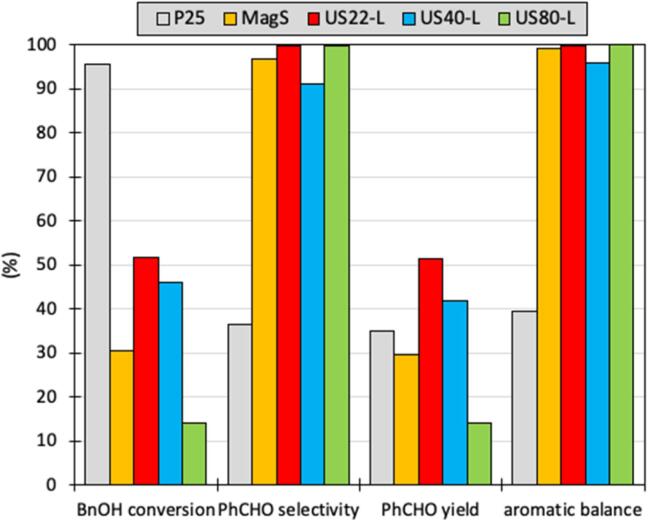
Photocatalytic performance of the photocatalytic oxidation of benzyl alcohol to benzaldehyde for the samples synthesized under ultrasound irradiation of different frequency (22, 40 and 80 kHz), in silent using magnetic stirrer and the commercial TiO_2_ P25, under UV light exposure for 6 h.

The US-assisted synthesized materials showed a wide deviation of photocatalytic BnOH conversion performance (Fig. 6) although they presented very high PhCHO selectivity. The first outcome regarding the effect of US frequency and power is that the materials obtained using low power US irradiation revealed higher photocatalytic performance as compared to the ones obtained by high power. Additionally, the samples obtained using 80 kHz as well as the samples of 22 and 40 kHz of high energy revealed very low BnOH conversion which was less than 14 % even after 6 h of light exposure. The best two performing lab-made materials were US22-L and US40-L, hence the discussion will focus on these two samples in comparison to the commercially and highly active P25, to the sample synthesized at low power 80 kHz (US80-L) and the one synthesized in silent. As can be seen from the BnOH conversion evolution, for all the samples (except P25) the conversion continuous with the same rate even up to 6 h. As can be seen from the results after 6 h of UV irradiation (Fig. 7), the best performing sample was US22-L, with the BnOH conversion to be 52 %, a value almost half comparing to P25 but ∼ 70 % higher than that of MagS sample. The photoluminescence (PL) spectra also support these results of photocatalytic conversion of BnOH, as the highest PL emission intensity was observed in case of MagS sample followed by US22-L, while P25 sample showed the least PL emission (Figure S6). P25 sample showed the highest photocatalytic conversion of BnOH followed by US22-L and MagS. The ultimately goal of this research work was to synthesized novel nanomaterials which posses higher selectivity toward the products in order to avoid the formation of any possible byproducts. As P25 showed the highest conversion of BnOH, but the selectivity was found least than the US22-L and MagS samples. Since the selectivity as well as the aromatic balance for all the synthesized materials was almost 100 %, comparing PhCHO yield is the best parameter for justification of the best photocatalyst. US22-L showed also the best yield of 51 %, a value that is 48 %, 74 %, 23 %, and 270 % higher compared to P25, MagS, US40-L, and US80-L samples, respectively.

One ultimate important feature of a heterogeneous catalyst is its recyclability/reusability. In order to study the shed light towards a potential effective reusability of the best performing synthesized materials, the photocatalytic partial selective oxidation of BnOH to PhCHO for selected samples i.e US22-L, MagS and P25 was studied under the same experimental conditions. The results of reusability studied, presented in Figure S7, revealed that all these selected samples exhibited an almost stable photocatalytic selective oxidation of BnOH efficiency for up to the 5 runs. The leaching tests after the 5th run of the photocatalytic experiment were performed by energy dispersive X-ray fluorescence (EDXRF). The results of EDXRF spectrum were collected Figure S8 and did not show any peak corresponds to Ti. Hence, we can conclude that no metal leaching takes place even after 5 consequent photocatalytic cycles, with the photocatalytic efficiency to be stable.

**Fig. 8:**
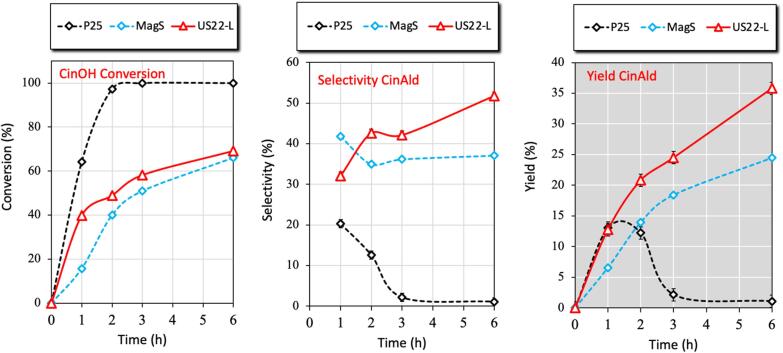
The conversion of Cinnamyl alcohol (CinOH) as well as the selectivity and yield to the partial oxidized product cinnaldehyde (CinAld) by TiO_2_ P25, US22-L and MagS samples under UV light irradiation for up to 6 h.

In order to determine if the ultrasound assisted material can present the highest selectivity and yield for the oxidation of other biomass derived aromatic compound, cinnamyl alcohol (CinOH) was chosen. The results of CinOH conversion as well as the selectivity and the yield towards the partial oxidation product cinnaldehyde (CinAld) are collected in Fig. 8. As in the case of benzyl alcohol, the commercial benchmark TiO_2_ P25 showed the greatest and fastest CinOH conversion, but the selectivity to the desired aldehyde was very low and hence the yield also. After 2 h of reaction, the entire CinOH was converted but the yield of CinAld was only ∼ 12 %. Regarding the synthesized samples, the one obtained under the influence of ultrasound revealed faster CinOH conversion and more importantly, the CinAld selectivity and so also the yield were significantly higher comparing to the sample obtained under silent conditions. For US22-L, the CinOH conversion reached almost 70 %, with the CinAld selectivity and yield to be ∼ 50 and ∼ 35 %, respectively. Another crucial outcome noted after these tests was that benzyl aldehyde was also formed during the photocatalytic tests, with the results to be collected in Figure S9. P25 showed faster formation of benzaldehyde compared to the other two samples, but it showed the fastest decomposition too. In between US22-L and MagS samples, the former one showed once again significant faster formation of benzyl aldehyde.

Based on all the above presented physicochemical features and the photocatalytic results can be concluded that to develop and synthesize the proper photocatalyst for a specific selective oxidation process is complicated with many factors. The clear outcomes although are that the optimized utilization of ultrasound as a synthetic tool can be assumed as an effective and prosperous strategy on nanomaterials design. It should be always considered that US irradiation during the synthesis has a bimodal nature, since chemical and physical phenomena take place. The latter ones are responsible for the mixing of the suspension and can control the way of crystal growth. The chemical effects of US can affect the surface chemical heterogeneity. The most important physicochemical feature when compare the synthesized material with P25 is that all of them revealed a basic surface pH in the range of 7.9 to 8.9. This is in a good agreement with the use of strong base during the synthesis, which blocks the formation of surface acidic groups. The surface features are more important. As the XPS measurements showed that the all the synthesized TiO_2_ samples contained carbon residues originating from the organic precursors may be incorporated on the surface of the synthesized samples. Another US22-L sample showed higher oxygen content as compared to other synthesized and commercial TiO_2_ samples which suggested that the organic can be oxidize to carbonate or carboxylate. So the presence of organic residues containing the carbon and oxygen linkages may lead to the photosenstizer effect which can help to improve the light absorption and the transfer of photo generated electrons [24], [45]_._ Also the synthesized samples contained higher amount of sodium which may lead to higher surface basicity. It can be suggested based also on previous reports that the Lewis acidic surface functional groups can promote the conversion of the benzaldehyde, a fact which is not desired. The higher partial selective photocatalytic activity of US assisted synthesized samples can be linked to the morphological features as these samples showed the 1-D nanostructures which possessed the improved light harvesting of the samples. The another possible reason is the formation of cage of nanostructures can also improve the diffusion of BnOH molecule to the inner part of the sample which also led to the higher activity by increasing the higher contact area for this reaction. As the thermal analysis revealed that the higher weight loss suggested the presence of hydroxyl radicals or may be some other functional groups which can also improve this photocatalytic performance [31]. The formation of the amorphous with higher specific surface area or defecated nanomaterials also lead to higher photocatalytic performance as compared to crystalline nanomaterials [46], [47]. The presence of micropores and mesopores in the synthesized samples as showed by the nitrogen sorption measurements (Fig. 2a) may contained small bulk volume that can also help in the diffusion of photogenerated carriers [48]. Although, these parameters are not enough alone in order to achieve high BnOH and CinOH conversion, and PhCHO and CinAld selective formation. And in general, we can conclude that the utilization of ultrasound irradiation as a synthetic tool is a promising strategy towards novel nanophotocatalyst and further research is required in order to optimize the materials and to determine conclusively how the cavitation effect is responsible for the growth of the nanocrystals and nanoparticles.

## 4 Conclusions

The use of ultrasonication as a synthetic tool towards novel titanium oxide/titanate by precipitation revealed that the final physicochemical properties of the materials can be tuned and optimized by altering the frequency and/or the ultrasound power (US). An interesting outcome is that the use of US led to novel samples of significant high textural parameters (specific surface area up to 155 m^2^/g) and of a mix morphological and structural nature consisting of 1-D nano-structures (nanorods-like), nanolayers and not well-defined (amorphous) areas. On the contrary, the sample prepared in silent (without ultrasound exposure, MagS) revealed lower porosity and specifica surface area (124 m^2^/g) and spherical shaped particles. The samples synthesized using low power/amplitude US frequencies (22 or 40 kHz) showed the highest selective photoactivity for the partial oxidation of BnOH to PhCHO as compared to 80 kHz and MagS. In between the samples obtained using 22 or 40 kHz, the ones of 22 kHz showed slightly higher and more selective BnOH photo-oxidation efficiency, predominately due to the better defined nano structures and the higher specific surface area. Even more importantly, the sample obtained using 22 kHz sonication of low power showed the best performance towards the selective oxidation of another lignin derived model compound, cinnamyl alcohol. Last but not least, it can be suggested that the presence of the surface hydroxyl groups have a positive impact on the selective oxidation, since the best performing sample showed the highest ratio of –OH to Ti-O-Ti groups. Taking all into consideration, the present research work clearly indicated that the use and optimization (frequency and/or amplitude) of ultrasound exposure as a synthetic tool can lead to novel nanomaterials with specific physicochemical featured and enhanced/selective photo activity.

## Declaration of competing interest

The authors declare that there is no known competing interests or personal relationships that could have appeared to influence the research work reported in this manuscript.

## CRediT authorship contribution statement

**Abdul Qayyum:** Conceptualization, Investigation, Visualization, Formal analysis, Writing – original draft. **Dimitrios A. Giannakoudakis:** Conceptualization, Writing – original draft, Writing – review & editing. **Dariusz Łomot:** Conceptualization, Visualization. **Ramón Fernando Colmenares-Quintero:** Writing – review & editing. **Alec P. LaGrow:** Investigation, Formal analysis. **Kostiantyn Nikiforow:** Investigation, Formal analysis. **Dmytro Lisovytskiy:** Investigation, Formal analysis. **Juan Carlos Colmenares:** Writing – review & editing, Resources, Conceptualization, Project administration.

## Declaration of Competing Interest

The authors declare that they have no known competing financial interests or personal relationships that could have appeared to influence the work reported in this paper.
