# Supplementary material for: Tuning the physicochemical features of titanium oxide nanomaterials by ultrasound: Elevating photocatalytic selective partial oxidation of lignin-inspired aromatic alcohols
Source: Ultrason Sonochem. 2023 Jan 21;94:106306. doi: 10.1016/j.ultsonch.2023.106306 (PMC9894921; doi:10.1016/j.ultsonch.2023.106306)
Supplement: Supplementary data 1 [file mmc1.docx]

**Tuning the physicochemical features of titanium oxide nanomaterials by ultrasound: Elevating photocatalytic selective partial oxidation of lignin-inspired aromatic alcohols**

**﻿** Abdul Qayyum ^a,^*, Dimitrios A. Giannakoudakis ^a,^*, Dariusz Łomot ^a^, Ramon Fernando Colmenares-Quintero ^b,^*, Alec P. LaGrow ^c^, Kostiantyn Nikiforow ^a^, Dmytro Lisovytskiy ^a^, Juan Carlos Colmenares ^a,^*

^a^ Institute of Physical Chemistry, Polish Academy of Sciences, Kasprzaka 44/52, 01-224, Warsaw, Poland.

^b^ Faculty of Engineering, Universidad Cooperativa de Colombia, Medellín 50031, Colombia.

^c^ Scientific Imaging Section, Okinawa Institute of Science and Technology Graduate University, Kunigami-gun, Okinawa 904-0412, Japan.

**Experimental:**

The surface pH was measured by dispersing 100 mg of sample to 50 mL of mili Q water in a closed vessel. After 16 h of equilibration in the dark condition, the pH was measured.

Energy dispersive X-ray fluorescence analysis (EDXRF) of the solution (after filtration) after the 5^th^ run of photocatalytic experiments for the selected samples, was carried out using MiniPal 4 equipment from PANalytical Co, with a Rh-tube and silicon drift detector (resolution 145 eV). The spectra were collected in air atmosphere, without using of a filter, at a tube voltage of 30 kV in order to evaluate the presence of Titanium in (liquid) samples under investigation (to avoid leaching). The time of acquisition was set to 120 s and the tube current up to 30 μA.

The Photoluminescence experiments were performed at the excitation wavelength 310 nm by using an instrument Fluorolog FL3-22 (Horiba Jobin Yvon).

**
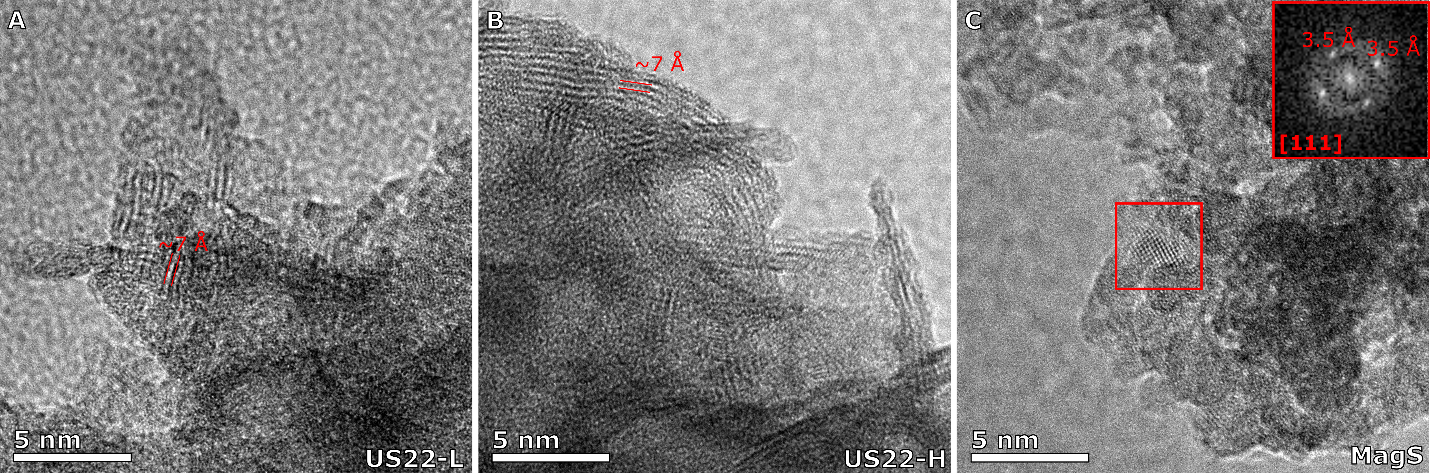
**

**Figure S1.** HR-TEM images of US22-L (a), US22-H (b) and MagS (c) samples.

**
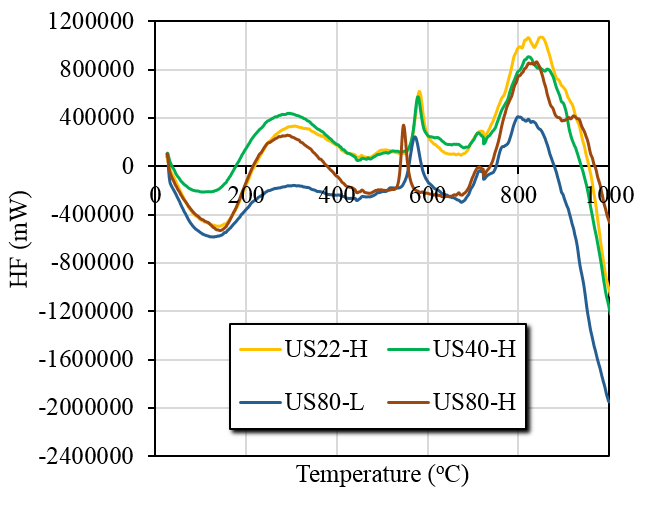
**

**Figure S2.** Differential thermal analysis for US assisted synthesized TiO_2_ samples_._


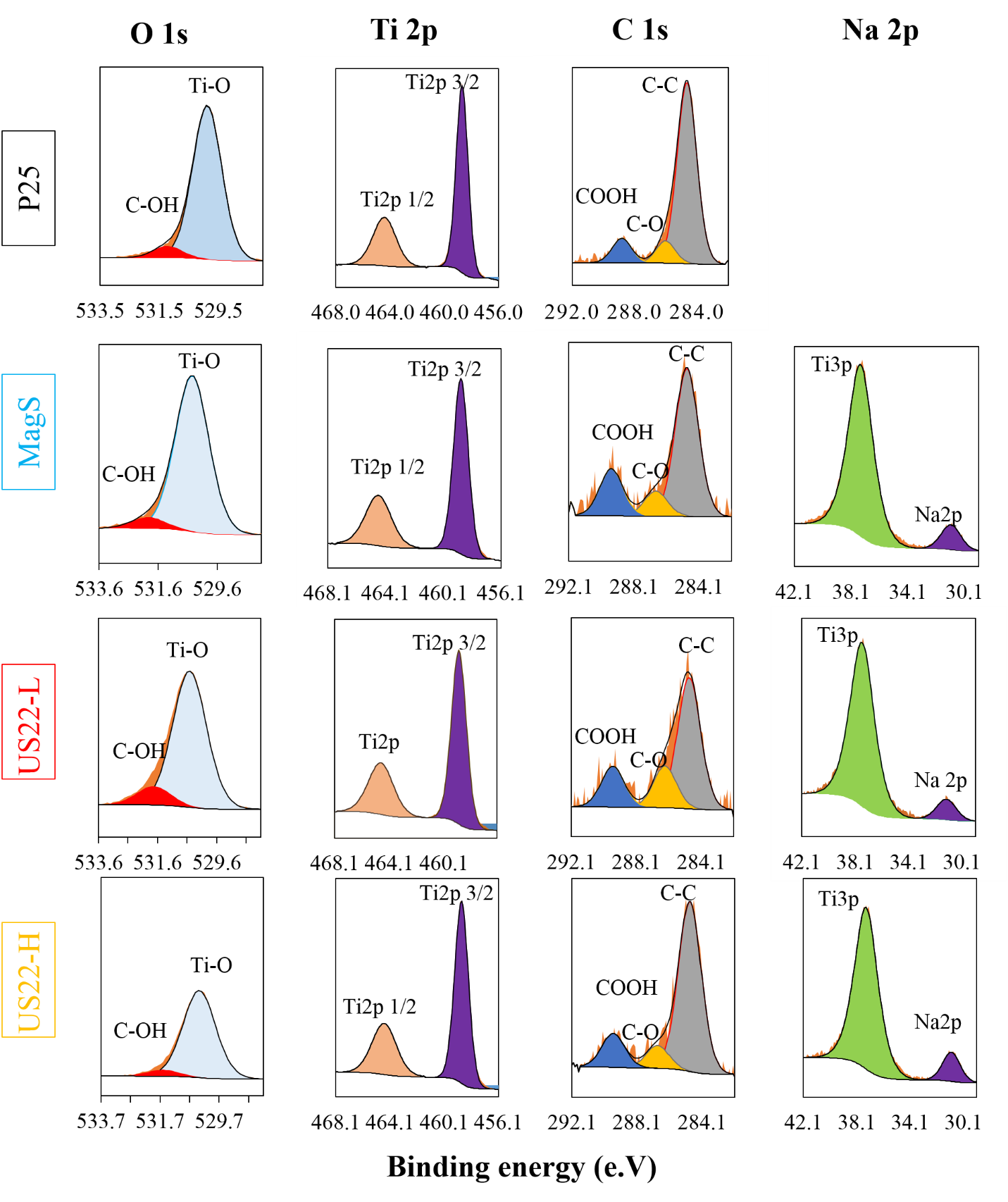


**Figure S3.** XPS high-resolution deconvoluted core energy level spectra of O 1s, Ti 2p, C 1s and Na 2p for P25, MagS, US22-L and US22-H samples.


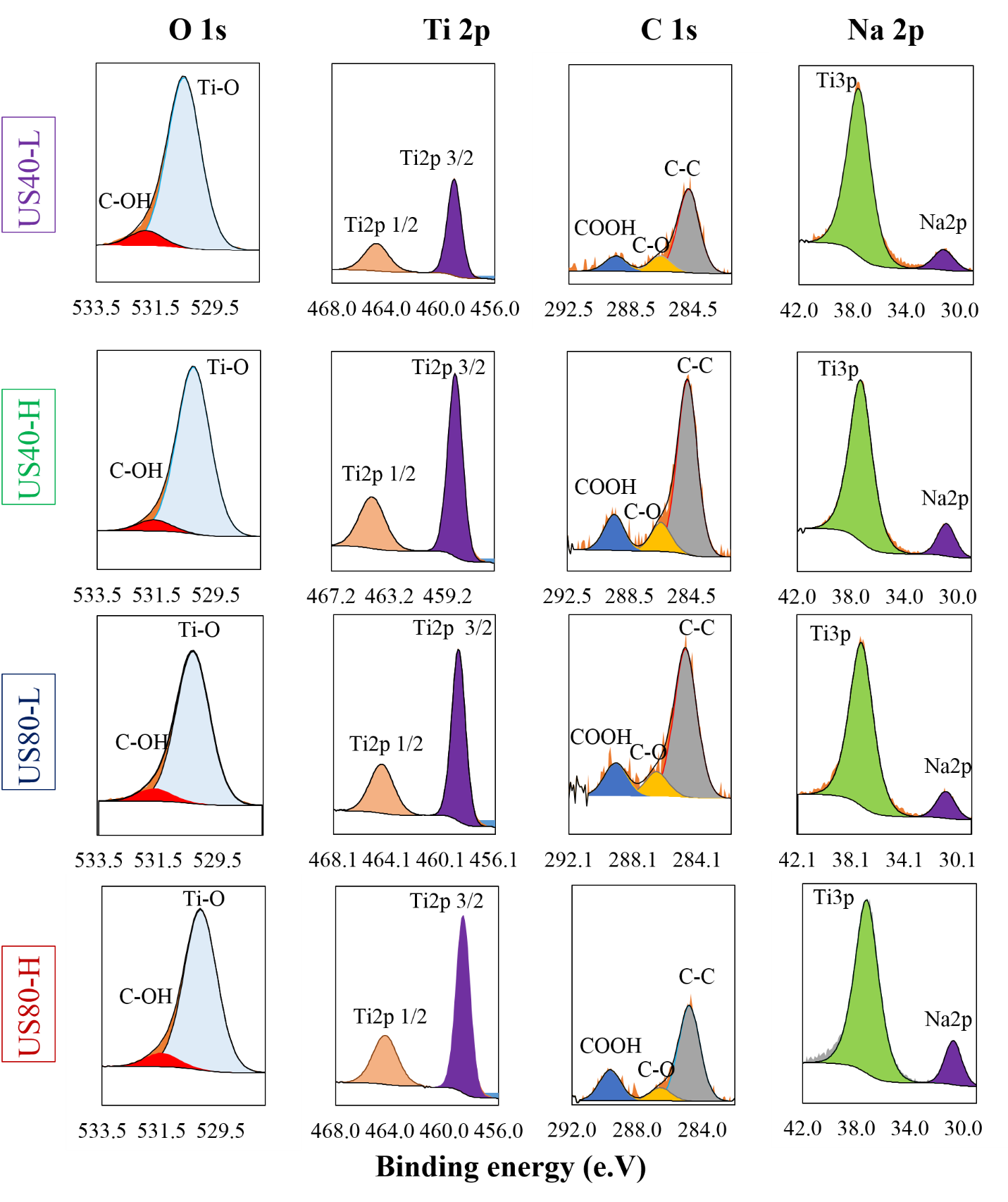


**Figure S4.** XPS high-resolution deconvoluted core energy level spectra of O 1s, Ti 2p, C 1s and Na 2p for US40-L, US40-H, US80-L and US80-H samples.


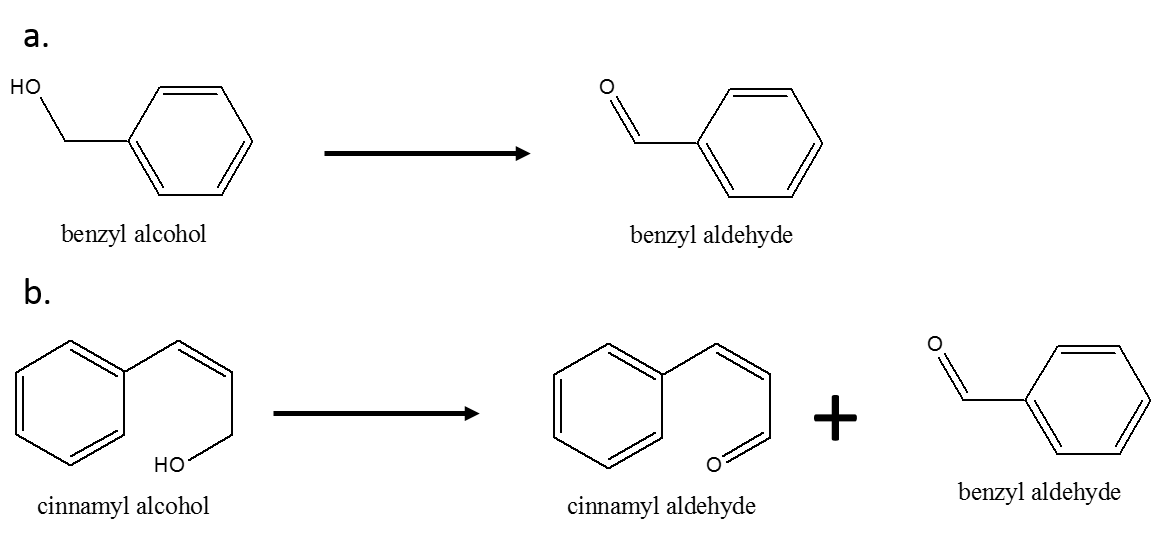


**Figure S5.** Photocataytic reaction of benzyl alcohol and cinnamyl alcohol to the corresponding product.

**Figure S6.** Photoluminescence emission spectra of US22-L, MagS and P25 samples.


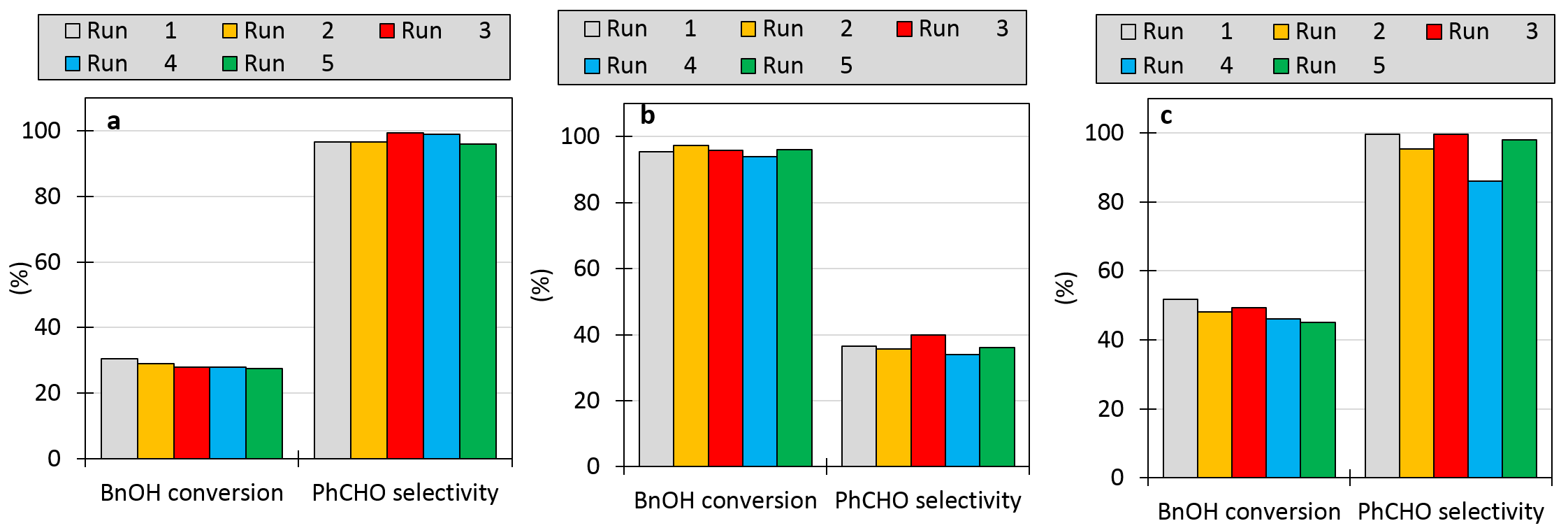


**Figure S7.** Reusability studied of the photocatalytic partial selective oxidation of benzyl alcohol to benzyl aldehyde by using catalyst MagS (a), P25 (b) and US22-L (c).


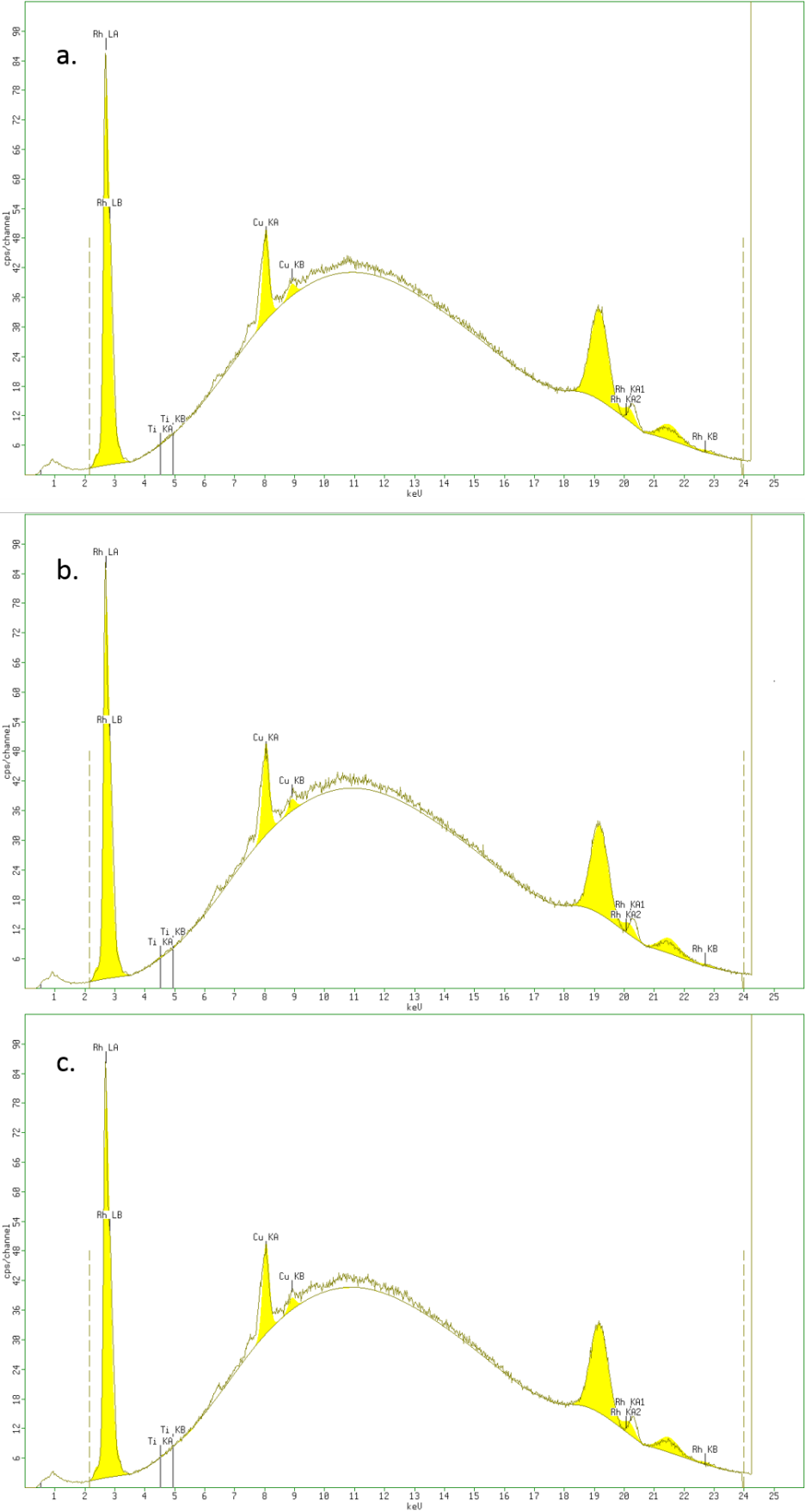


**Figure S8.** XRF measurement of the solution after the 5^th^ run of photocatalytic experiments by using samples P25 (a), MagS (b) and US22-L (c).


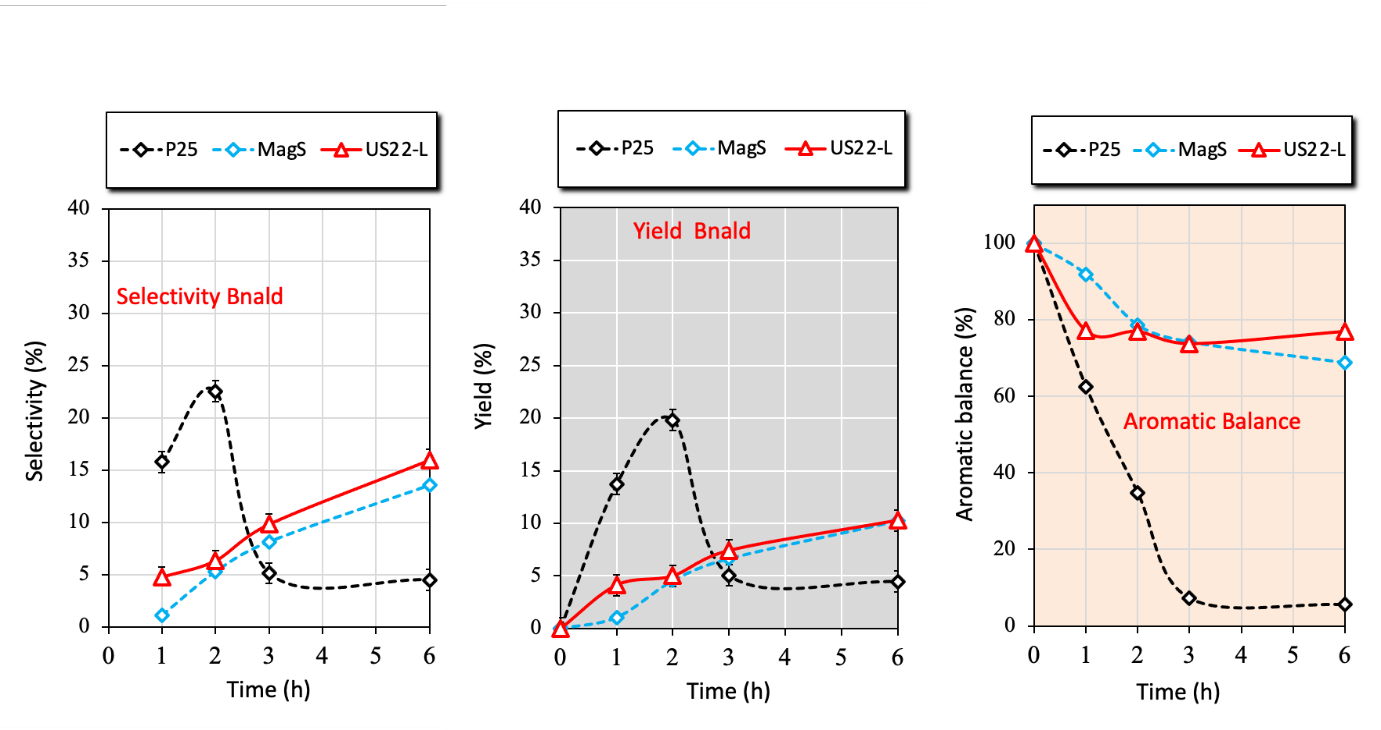


**Figure S9.** The selectivity and yield of benzaldehyde recorder during the photocatalytic tests of cinnamyl alcohol (CinOH) using TiO2 P25, US22-L, and MagS under UV light irradiation for up to 6 hours.
